# Neural correlates of socio-emotional perception in 22q11.2 deletion syndrome

**DOI:** 10.1186/s11689-018-9232-2

**Published:** 2018-04-10

**Authors:** Lydia Dubourg, Pascal Vrticka, Martin Debbané, Léa Chambaz, Stephan Eliez, Maude Schneider

**Affiliations:** 1 Developmental Imaging and Psychopathology Laboratory, Department of Psychiatry, School of Medicine, University of Geneva, Campus Biotech, Chemin des mines 9, 1202 Geneva, Switzerland; 2 Center for Contextual Psychiatry, Department of Neurosciences, Research Group Psychiatry, KU Leuven, Leuven, Belgium; 3 Department of Social Neuroscience, Max Planck Institute for Human Cognitive and Brain Sciences, Leipzig, Germany; 4 Developmental Clinical Psychology Research Unit, Faculty of Psychology and Educational Sciences, University of Geneva, Geneva, Switzerland; 5 Research Department of Clinical, Educational and Health Psychology, University College London, London, UK; 6 Department of Genetic Medicine and Development, School of Medicine, University of Geneva, Geneva, Switzerland

**Keywords:** 22q11.2 deletion syndrome, Socio-emotional perception, Default mode network, fMRI

## Abstract

**Background:**

Social impairments are described as a common feature of the 22q11.2 deletion syndrome (22q11DS). However, the neural correlates underlying these impairments are largely unknown in this population. In this study, we investigated neural substrates of socio-emotional perception.

**Methods:**

We used event-related functional magnetic resonance imaging (fMRI) to explore neural activity in individuals with 22q11DS and healthy controls during the visualization of stimuli varying in social (social or non-social) or emotional (positive or negative valence) content.

**Results:**

Neural hyporesponsiveness in regions of the default mode network (inferior parietal lobule, precuneus, posterior and anterior cingulate cortex and frontal regions) in response to social versus non-social images was found in the 22q11DS population compared to controls. A similar pattern of activation for positive and negative emotional processing was observed in the two groups. No correlation between neural activation and social functioning was observed in patients with the 22q11DS. Finally, no social × valence interaction impairment was found in patients.

**Conclusions:**

Our results indicate atypical neural correlates of social perception in 22q11DS that appear to be independent of valence processing. Abnormalities in the social perception network may lead to social impairments observed in 22q11DS individuals.

**Electronic supplementary material:**

The online version of this article (10.1186/s11689-018-9232-2) contains supplementary material, which is available to authorized users.

## Background

The 22q11.2 deletion syndrome (22q11DS), also known as DiGeorge or velocardiofacial syndrome (VCFS), is a neurogenetic disorder affecting approximately 1 in 4000 live births [1] and occurring in up to 1:1000 pregnancies [2]. The syndrome is associated with elevated risk for schizophrenia [3] and is characterized by high rates of distinct positive and negative symptoms. It is well established that social dysfunction is also a common feature of the 22q11DS profile [4–6]. Individuals with 22q11DS are described as shy, withdrawn, and presenting social interaction problems particularly with their peers [4–6]. Up until now, only a few neuroimaging studies have investigated the neural correlates of socio-cognitive processes in the 22q11DS population to better understand the emergence of social deficits associated with the syndrome [7–9]. The neural substrates of social-emotional impairments hence remain largely unclear. In the current study, we therefore focused on the neural correlates of socio-emotional processes to extend our understanding of impairments in the 22q11DS population related to such mechanisms.

Social perception encompasses the processing of various social cues that individuals encounter in everyday life (e.g. face and voice). Social cue perception has been mostly investigated through face perception. In healthy individuals, face perception is mainly associated with increased activity in the fusiform face area, visual extrastriate cortex, lateral occipital gyri, anterior temporal pole, and posterior superior temporal gyrus [10, 11]. Moreover, the processing of emotional information in faces involves a similar network as emotion perception (see below), including limbic regions, inferior frontal gyrus, medial prefrontal gyrus, and putamen [11–13].

Until recently, most studies in patients with schizophrenia focused on higher order processes of social cognition (e.g. theory of mind) while lower aspects as social perception have received less attention. To our knowledge, only one study has examined brain activations in response to simple visual social cues [14]. This study reported abnormal neural activity during processing of social information. Hypoactivation in regions associated with visual processing (occipital and temporal regions) and increased cingulate activity during the processing of social vs. non-social images has been observed in patients with schizophrenia compared to controls. In patients with 22q11DS, as in schizophrenia, most studies have focused on higher-order socio-cognitive processes, highlighting impairments in theory of mind and emotion recognition [15, 16], and few functional magnetic resonance imaging (fMRI) studies have been conducted on this topic [7–9]. Andersson et al. [8] investigated social perception and reported hypoactivation of the fusiform in response to faces versus houses. In light of the high prevalence of social impairments in 22q11DS, additional studies examining the neural substrates of social perception and their link with social functioning are clearly needed.

A second aspect of social cognition that will be investigated in this study is emotion processing. Commonly, these processes are investigated while individuals are viewing pleasant or unpleasant images compared to neutral images. In healthy participants, a set of regions has been related to emotion processing, mainly including the limbic system (amygdala, anterior hippocampus, anterior insula, and cingulate gyrus), as well as brain stem nuclei, thalamus, ventral striatum, medial prefrontal cortex, posterior cingulate cortex, precuneus, lateral temporal cortex, and temporal pole [17, 18]. However, up until now, only three studies have investigated the neural basis of emotion processing in 22q11DS [7–9]. van Amelsvoort et al. [7] compared brain activity during the presentation of mixed emotional facial expressions between eight individuals with 22q11DS and nine controls. Two types of facial emotions (happy or angry) and neutral faces were presented in a block design. Patients with 22q11DS showed less activation in the right insula and frontal regions and more activation in occipital regions compared to controls. However, this study used a block design (with mixed emotion), which made it impossible to differentiate neural responses as a function of the type of valence. Two additional studies used only negatively valenced stimuli [8, 9] and confirmed the hypoactivation in regions related to emotion processing (superomedial prefrontal cortices) in patients with 22q11DS. However, and contrary to van Amelsvoort et al. [7], some regions involved in socio-cognitive processing (fusiform, anterior cingulate cortex) were also hypoactivated. Moreover, only one study investigated the relationship between brain activation and social functioning in individuals with 22q11DS [9]. The authors found that decreased brain activation during emotion perception was related to social difficulties in patients with 22q11DS. So far, studies investigating emotional processing in participants with 22q11DS reported results from very small samples (8 to 15 patients) and only one type of valence was explored. Consequently, the neural bases of emotion processing still remain unclear in this population, and the distinction between positive and negative emotion perception has never been properly investigated.

Finally, it is important to take into consideration that emotions can influence social processing, as it appears that there is an overlap between social information and emotion processing. Indeed, it has been argued that social cues are inherently emotional and that social information processing will therefore also involve emotion-processing networks [19]. To test this hypothesis, several studies in healthy individuals have been conducted and confirmed an interactive processing of social content and valence [19–21]. While a social content × valence interaction effect was found in the thalamus, superior temporal sulcus, middle orbito-temporal cortex, as well as in the anterior insula and lateral medial prefrontal cortex in several studies, Norris et al., Scharpf et al., and Vrtička et al. [19–21] also observed such interaction effect in the amygdala, fusiform gyrus, anterior superior frontal gyrus, and middle occipital cortex. Conversely, in the 22q11DS, the influence of valence on social and non-social information processing is currently unknown.

This study examined neural correlates of socio-emotional processing patients with 22q11DS compared to healthy controls. First, we investigated neural correlates of social perception. In line with the literature in schizophrenia [14], we expected to observe altered activations in the social perception network in participants with 22q11DS. Secondly, we examined neural response to positively and negatively valenced stimuli. Based on previous studies in participants with 22q11DS, we expected to observe a significant decrease in regions involved in emotion (e.g. insula, frontal regions) and socio-cognitive processing (e.g. fusiform). Thirdly, in order to investigate the influence of emotions on social perception in 22q11DS, we also examined a social content × valence interaction. Finally, we hypothesized that impaired activation of socio-emotional networks would be related to socio-cognitive deficits in participants with 22q11DS.

## Methods

### Participants

Participants were recruited through parent associations or word of mouth and were tested in our research laboratory during an ongoing longitudinal study. Twenty-two participants with 22q11DS aged between 12 and 32 were included (mean age = 20.3 ± 5, 17 (77%) females). The presence of a 22q11.2 microdeletion was confirmed in all participants using quantitative fluorescent polymerase chain reaction (QF-PCR). Patients diagnosed with a DSM-IV psychotic disorder were excluded from this study (*N* = 2) to decrease the influence of confounding factors on brain activation patterns (e.g. long-term use of antipsychotics). However, some patients met formal diagnostic criteria for other current psychiatric conditions, and 10 participants were under medication at the time of testing (see Table 1). Furthermore, 22 controls including siblings (*n* = 13) and unrelated individuals (*N* = 9) aged between 12 and 32 (mean age = 19.7 ± 5, 15 (68%) females) were also included and were screened for the presence of any neurological problems, and psychological or learning difficulties (see Table 1).

**Table 1:** Demographic informations for 22q11DS and healthy control participants

			Diagnostic group		Comparison	
			22q11DS	Controls	ANOVA	*p* value
*N*			22	22		
Age			20.3 (±5.2)	19.7(±5.1)	0.120	0.730
Gender (% of female)			17 (77%)	15 (68%)	0.442	0.510
Full IQ (mean (SD)) Benton face recognition			74 (±12)	118 (±11)	162.8	< 0.01
			39.4 (3.9)	47.6 (2.9)	60.1	0.001
SRS (mean (SD))*		SRS awareness	58 (±12)	47 (±9)	7.4	0.001
		SRS cognition	57 (±9)	45 (±6)	17.4	< 0.001
		SRS communication	58 (±11)	45 (±5)	20.4	< 0.001
		SRS motivation	60 (±12)	44 (±6)	18.1	< 0.001
		SRS RRB	58 (±11)	45 (±5)	20.4	< 0.001
		SRS Total	51 (±12)	44 (±6)	18.1	< 0.001
Psychiatric diagnosis (*N* (%))		Major depression disorder	5 (23%)			
		Specific phobia	3 (13%)			
		Simple phobia	1 (4%)			
		Generalized anxiety disorder	3 (13%)			
		Obsessive compulsive disorder	1 (4%)			
		Alcohol dependence	1 (4%)			
		Oppositional defiant disorder	1 (4%)			
		Delusions	2 (8%)			
		ADHD	3 (13%)			
Psychotropic medication	Categories	Antipsychotics	1 (4%)			
		Antidepressants	7 (32%)			
		Methylphenidate	4 (18%)			

*For the SRS, data were missing for five 22q11DS participants and six controls

Written informed consent was obtained from participants and their parents under protocols approved by the Swiss Ethics Committee on research involving humans.

### Clinical assessment

The presence of psychiatric disorders was assessed in adolescents below 18 years using the Diagnostic Interview for Children and Adolescents – Revised [22], and the mood and psychosis supplement of the Kiddie-Schedule for Affective Disorders and Schizophrenia Present and Lifetime version (K-SADS-PL; [23]). Adult participants were screened using the Structured Clinical Interview for DSM-IV axis disorders (SCID-I; [24]). Participants were also screened for attenuated positive and negative symptoms using the Structured Interview for Psychosis-risk Syndromes (SIPS; [25]). Symptoms are assessed on a 7-point severity scale (ranging from 0 to 6).

### Intellectual functioning

All participants completed the Wechsler Intelligence Scale for Children III or IV (WISC-III-R or WISC-IV-R; [26, 27]) or Adult III or IV (WAIS III or WAIS IV; [28, 29]) in order to obtain an evaluation of global intellectual functioning. Mean full-scale IQ was 74 (SD = 12) in participants with 22q11DS and 118 (SD = 11) in controls.

### Socio-cognitive measures

We also assessed socio-cognitive functioning in all participants. To do so, we administered the Benton Facial Recognition Test (BFRT; [30]), a measure of face recognition ability. Participants were asked to match non-emotional unfamiliar faces. They were presented one target and six other black and white faces (male or female). First, participants were asked to match the target face with an identical photo. Secondly, they had to match the target face with three photos taken from different angles or different lighting conditions. The total number of correct answer was used as main measure. Scores ranging from 41 to 54 indicate normal performance, from 39 to 40 borderline performance, between 37 and 38 moderate impairment, and below 37 severe impairment.

We also administered the second edition of the Social Responsiveness Scale (SRS-2; [31]), a measure of social functioning to parents of 17 participants with the 22q11DS and 16 controls to identify the presence and severity of social impairments. Data were missing for 11 individuals. The SRS is a 65-item parent questionnaire investigating the child’s social behaviour in the past 6 months. A 4-point Likert scale (0 = not true, 1 = sometimes true, 2 = often true, 3 = always almost true) is used to rate how often the child engages in the respective behaviour. The SRS provides information about five domains: social awareness, social cognition, social communication, social motivation, and restricted interests and repetitive behaviour. Raw scores for each scale are converted to a gender-specific T score representing the individual’s social behaviour impairment in each of the five domains. The five scales are summed and converted into a T score, resulting in an overall composite SRS score. T scores up to 59 are within the normal range, from 60 to 65 indicate mild deficiencies of reciprocal social behaviour, from 66 to 75 moderate impairment, and *≥*76 severe impairment.

### fMRI paradigm: design and procedure

**Fig. 1:**
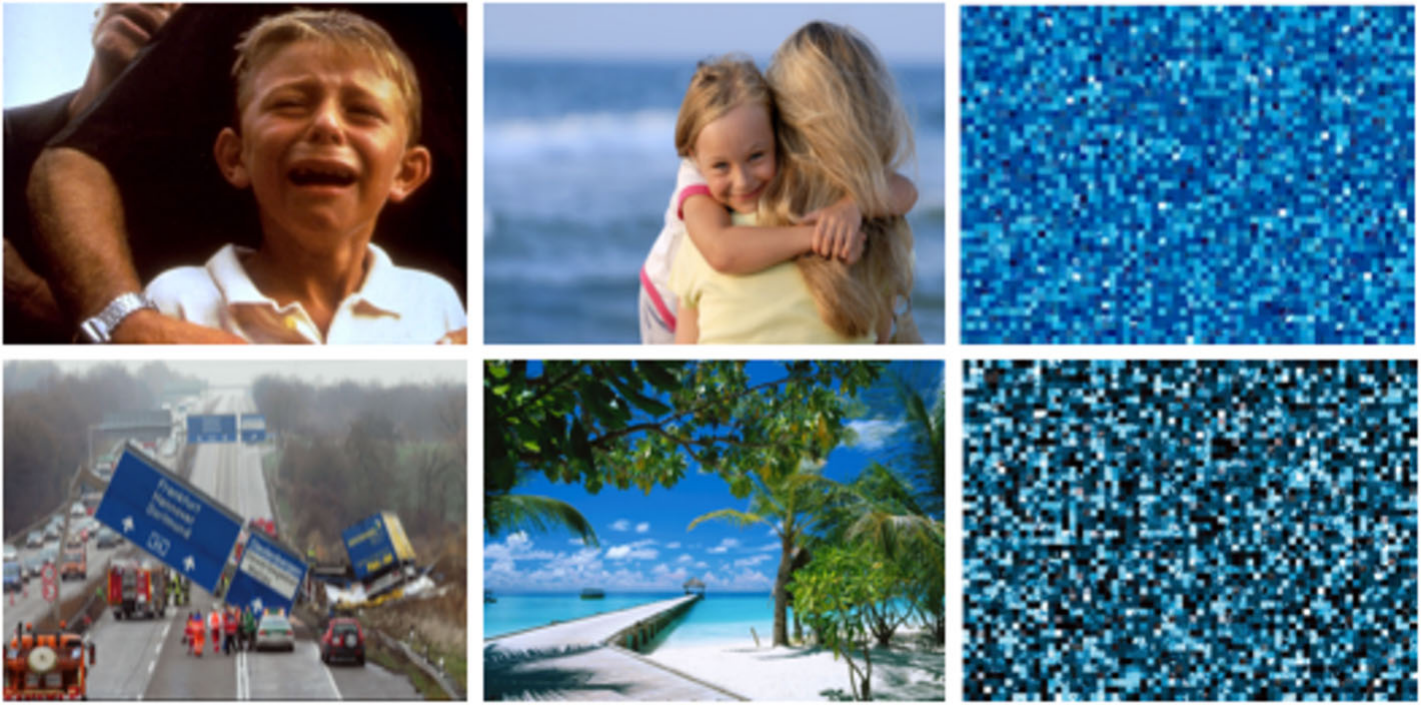
Example of stimuli. Left of right: negative, positive, and blurred. Top panel: social, bottom panel: non-social

The experiment consisted of two runs during which participants were presented with images from the International Affective Picture System (IAPS; [32]) and scrambled images. The scrambled images were created using the selected IAPS pictures (obtained using the Photoshop plugin: [33]. Participants were instructed to indicate whether the image was intact or scrambled using an MRI-compatible response box. Target images were divided into four categories using a 2 (social content) × 2 (valence) factorial design: social and positive valence, social and negative valence, non-social and positive valence, and non-social and negative valence. Positive and negative images were selected to elicit similar arousal levels (see Additional file 1: Table S1). Social images were defined as pictures that contained at least two human beings, while non-social images did not include any humans. During each run, 40 images (10 from each category) and 40 scrambled images were presented for 2 s with inter-trial intervals varying between 2500 and 5000 ms (Fig. 1). The presentation order of the two runs was counterbalanced between participants.

### Behavioural analysis

Between- and within-group comparisons of reaction time in response to the different conditions (social, non-social, positive, negative, scrambled) as well as accuracy were examined using univariate ANOVAs in SPSS Version 21.

### fMRI data acquisition and analysis

Structural and functional images were acquired using a Siemens Prisma 3 T scanner at the Geneva Center for Biomedical Imaging (CIBM). The acquisition protocol for the structural sequence was a 3D volumetric pulse sequence with TR = 2500 ms, TE = 3 ms, flip angle = 8°, acquisition matrix = 256 × 256, field of view = 22 cm, slice thickness = 1.1 mm, and 192 slices. The fMRI acquisition consisted of two sequences of 8 min each and resulting in 430 blood-oxygenation-level-dependent (BOLD) images (TR = 2200 ms, echo time, TE = 30 ms, 36 axial slices, slice thickness = 4.0 mm, spacing between slices = 2.5 mm, flip angle = 85°, field of view [FOV] = 235 mm).

fMRI data were processed and analysed using Statistical Parametric Mapping 12 (SPM12; Welcome Department of Neuroscience, London UK). Functional images were realigned using rigid body registration and resliced. Participants with motion exceeding 3 mm in any of the six directions were excluded from the analyses (four patients and two controls).

Each participant’s structural image was coregistered to the mean of the realigned functional images and segmented with the Dartel option to obtain tissue classification. Finally, we normalized to 1 mm^3^ Montreal Neurologic Institute (MNI) space and spatially smoothed with a 6-mm at full-width half-maximum three-dimensional Gaussian kernel, employing the diffeomorphic anatomical registration using exponential lie algebra algorithm (DARTEL).

For each participant, brain responses were analysed in the context of the general linear model (GLM) approach using a two-level procedure. At the first level, the five experimental conditions (social positive, social negative, non-social positive, non-social negative, and scrambled) were entered in the model. The six movement parameters were included as additional regressors of no interest in the design matrix. Changes in the BOLD signal were obtained using the estimated GLM parameters for each contrast of interest (social vs. non-social, social vs. scrambled, non-social vs. scrambled, positive vs. negative, positive vs. scrambled, negative vs. scrambled) and interaction contrasts (social content × valence). In the second-level analysis, we first computed the overall effect per group for each contrast; then, individual contrast images were included in an independent two-sample *t*-test to determine significant brain activation in patients with 22q11DS versus healthy controls. In order to account for the possible influence of age, sex, IQ, and handedness on brain activation, these four variables were entered as control covariates in the model for the second-level analysis (see Additional file 1 for a description of the results without IQ as covariate). Moreover, as medication could also be an important confound in this study, the presence or absence of medication was added as covariate in all the analyses.

For the within- and between-group comparisons, we chose a primary voxel-level statistical threshold of *p* < 0.001 (uncorrected, whole brain) and *k* ≥ 20. For clusters that survived this threshold at the voxel level, a cluster-extent family-wise correction (FWEc) for multiple comparisons at *p* < 0.05 was applied. Neuroanatomical locations of activations were identified using Talairach Daemon software after adjusting coordinates to allow differences between the MNI and Talairach templates [34].

To investigate potential associations between brain activity differences of the main contrasts and clinical variables, we first extracted raw activations (betas) from significant clusters and a priori ROIs (see below). Then, using multiple regression analyses, we tested these betas against face recognition and social functioning scores with age, gender, handedness, and IQ as covariates. Secondly, we performed whole-brain multiple regression analyses. Each regression included one contrast (i.e. social vs. non-social), included the covariates of interest (socio-cognitive measures), and was controlled for age, sex, handedness, and IQ. Effects of covariates on brain activations were investigated in both directions. A combined statistical threshold of *p* < 0.001 uncorrected at the peak and *p* < 0.05 FWE-corrected at the cluster level was applied.

## Results

### Behavioural results

Reaction time values are provided in Table 2. Within both groups, reaction times were faster for scrambled versus intact images (*p* < .05). However, no significant differences were present for intact images regarding social content (social/non-social) and valence (positive/negative) (*p* > .05). Conversely, between-group comparisons revealed that 22q11DS participants had significantly faster reaction times compared to controls in all experimental conditions except for scrambled images (*p* < .05). Comparison of accuracy within and between groups did not reveal any significant differences (*p* > 0.05).

**Table 2:** Reaction times for 22q11DS (22q) and healthy control (HC) participants

Condition	Reaction times (ms)	
	22q (M ± SD)	HC (M ± SD)
Social	818.4 ± 178.2	834.8 ± 178.7
Non-social	846.3 ± 147.5	945.43 ± 551.5
Positive	833.1 ± 153.6	926.5 ± 551.7
Negative	831.5 ± 171.2	853 ± 189.1
Scrambled	711.9 ± 129.6	700.55 ± 130.6

### Neuroimaging results

#### Social perception

##### Social versus scrambled contrast

We first determined brain activation in response to social stimuli (social vs. scrambled trials). In the control group, this contrast revealed enhanced activation of the left postcentral gyrus and inferior parietal lobule (see Table 3). In participants with 22q11DS, the same contrast did not show any significant activation, and the between-group comparison did not return any significant difference either.

**Table 3:** Brain regions showing significant increase in social perception contrasts within and between groups

*k*	*t*	MNI (*x*,*y*,*z*)			Hemisphere	Region	BA
*Healthy controls*							
Social > Scrambled							
1095	5.36	−40	−39	49	L	Inferior parietal lobule	40
	5.26	−47	−34	50	L	Postcentral gyrus	2
	4.43	−52	−25	50	L	Postcentral gyrus	2
	4.35	−50	−47	56	L	Inferior parietal lobule	40
	4.17	−47	− 42	56	L	Inferior parietal lobule	40
	4.16	− 41	−47	56	L	Inferior parietal lobule	40
	3.69	−53	−22	53	L	Postcentral gyrus	
Nonsocial > Scrambled							
4239	5.92	−13	−103	8	L	Cuneus	18
	5.34	−6	−100	14	L	Middle occipital gyrus	18
	5.33	−4	−94	23	L	Cuneus	19
1678	6.00	−26	−71	−7	L	Lingual gyrus	19
		−22	−73	−14	L	Lingual gyrus	18
		−9	−83	−11	L	Lingual gyrus	18
1244	7.86	12	−97	18	R	Cuneus	17
	5.65	13	−89	−7	R	Lingual gyrus	17
	4.76	16	− 96	−2	R	Cuneus	17
622	6.10	21	−63	13	R	Posterior cingulate	31
		17	−62	4	R	Posterior cingulate	30
Social > Non-Social No cluster pFWEc < 0.05							
*22q11DS patients*							
Social > Scrambled No cluster pFWEc < 0.05							
Nonsocial > Scrambled							
1416	6.45	−13	−91	8	L	Cuneus	17
	5.70	−5	− 97	7	L	Cuneus	18
	5.68	−11	−96	5	L	Cuneus	17
	5.26	−9	−100	12	L	Middle occipital gyrus	17
	4.65	−17	− 93	17	L	Middle occipital gyrus	18
	4.35	−13	−95	17	L	Cuneus	18
675	5.62	−28	−41	72	L	Postcentral gyrus	2
	4.96	−22	−40	73	L	Postcentral gyrus	2
	4.53	−26	−39	62	L	Postcentral gyrus	3
Social > Non-Social No cluster pFWEc < 0.05							
*Group comparison*							
Social > Scrambled No cluster pFWEc < 0.05							
Non-social > Scrambled No cluster pFWEc < 0.05							
Social > Non-Social CTRL > VCFS							
1683	5.14	0	46	20	L	Medial frontal gyrus	9
	4.93	−11	51	14	L	Medial frontal gyrus	10
	4.26	−11	39	18	L	Anterior cingulate	32
	4.22	−19	41	15	L	Anterior cingulate	32
	4.18	−11	43	19	L	Medial frontal gyrus	9
	4.11	−7	46	21	L	Medial frontal gyrus	9
	4.09	−13	45	9	L	Anterior cingulate	32
	3.92	−5	50	30	L	Medial frontal gyrus	9
	3.77	−11	41	11	L	Anterior cingulate	32
	3.72	−6	45	11	L	Anterior cingulate	32
	3.41	−7	53	28	L	Superior frontal gyrus	9
1466	5.71	52	−61	39	R	Inferior parietal lobule	40
	5.05	52	−57	38	R	Inferior parietal lobule	40
	4.63	47	−58	35	R	Supramarginal gyrus	40
908	4.80	−26	27	41	L	Middle frontal gyrus	8
	4.75	−25	29	45	L	Middle frontal gyrus	8
	3.98	−33	25	41	L	Middle frontal gyrus	8
	3.97	−16	34	45	L	Superior frontal gyrus	8
	3.74	−25	43	34	L	Superior frontal gyrus	9
	3.66	−21	37	43	L	Superior frontal gyrus	8
	3.53	−25	41	38	L	Middle frontal gyrus	9
659	4.37	42	20	40	R	Precuneus	9
	4.23	41	20	44	R	Middle frontal gyrus	8
	4.16	34	23	49	R	Middle frontal gyrus	6
	3.82	34	2	39	R	Middle frontal gyrus	6
	3.79	29	20	37	R	Middle frontal gyrus	9
	3.54	36	26	43	R	Middle frontal gyrus	8
551	4.67	13	−43	34	R	Posterior cingulate	31

*Abbreviations*: *k* cluster size, *t* t scores, *MNI* Montreal Neurological Institute, *BA* Brodman Area

##### Non-social versus scrambled contrasts

Similar analyses were performed to determine regions activated during non-social image perception. Separate contrasts in each group revealed that controls showed significant activation to non-social images in bilateral cuneus, lingual gyrus, left middle occipital gyrus, and right posterior cingulate, whereas in participants with 22q11DS, non-social images significantly activated the left cuneus, middle occipital gyrus, and left postcentral gyrus. Finally, direct between-group comparison did not reveal any significant difference.

##### Social versus non-social contrast

**Fig. 2:**
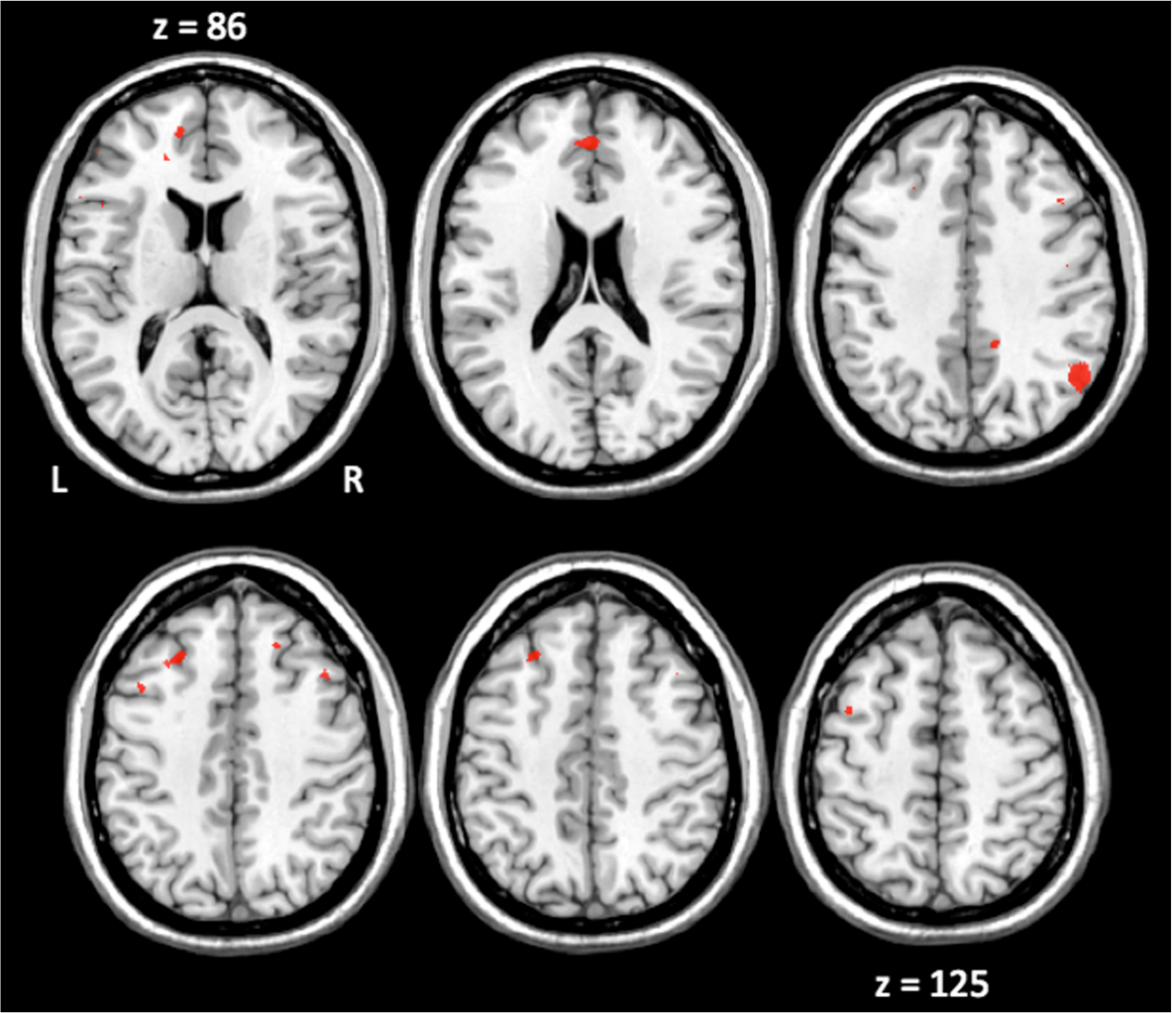
Increased activation in healthy participants compared to patients with 22q11DS for the condition Social > Non-social. Activations are reported in Table 3. (L = left, R = right)

Next, we tested brain activation specifically associated with social perception (social vs. non-social images). No significant pattern of activation was observed in the within-group comparisons. However, the direct between-group comparison showed greater activation in controls compared to individuals with 22q11DS in the bilateral medial frontal gyrus, left anterior cingulate, middle frontal gyrus, and superior frontal gyrus, as well as in the right supramarginal gyrus, inferior parietal lobule, precuneus, and posterior cingulate (see Fig. 2 and Table 3).

In previous studies conducted in healthy controls using similar images, the same contrast (social > non-social images) elicited a much wider network of activation, notably including the bilateral fusiform gyrus, bilateral amygdala, and superior temporal sulcus [35]. We consequently conducted a post hoc analysis for these regions of interest. Using Marsbar [36], spheres with a 6-mm radius were defined around the center of these regions of interest bilaterally. Beta values per individual and per condition were extracted, averaged, and used for ANOVAs and *t*-tests in SPSS. No significant differences within or between groups for these three ROIs were observed.

#### Emotion processing

##### Positive emotional processing

To investigate brain activation related to the processing of positive emotional stimuli, we then contrasted positive vs. scrambled images. The within-group contrast revealed lingual gyrus activation in both groups. Conversely, increased activation to positive images was found in the posterior cingulate and cuneus only in healthy controls, while increased activation to positive images was observed in the precuneus, postcentral gyrus, and superior and inferior parietal lobule only in patients (see Table 4). Nonetheless, the direct between-group comparison did not show any significant differences between controls and 22q11DS participants.

**Table 4:** Brain regions showing significant increase in emotional experience contrasts within and between groups

*k*	*t*	MNI (*x,y,z*)			Hemisphere	Region	BA
*Healthy controls*							
Positive > Scrambled							
2639	5.08	−8	−77	17	L	Cuneus	18
	5.05	−14	−77	8	L	Cuneus	17
	4.79	−12	−60	0	L	Lingual gyrus	18
1472	5.10	−18	−91	26	L	Cuneus	18
	5.09	−6	−95	19	L	Cuneus	18
894	7.16	21	−66	14	R	Posterior cingulate	31
	4.35	11	−62	12	R	Posterior cingulate	30
Negative > Scrambled							
1008	4.93	15	−90	−7	R	Lingual gyrus	17
	3.97	14	−98	1	R	Cuneus	17
877	5.59	−16	−70	6	L	Cuneus	30
	4.24	−10	−65	4	L	Cuneus	30
	4.15	−21	−61	3	L	Posterior cingulate	30
Positive > Negative No cluster FEW < 0.05							
*22q11DS patients*							
Positive > Scrambled							
1505	5.97	22	−54	55	R	Precuneus	7
	5.82	23	−60	50	R	Precuneus	7
	5.59	29	−62	60	R	Superior parietal lobule	7
	5.47	33	−56	63	R	Inferior Parietal Lobule	40
	5.10	26	−59	47	R	Superior parietal lobule	7
	4.95	23	−63	55	R	Precuneus	7
	4.31	26	−65	57	R	Superior parietal lobule	7
1066	6.38	−25	−41	70	L	Postcentral	2
	5.84	−34	−46	65	L	Postcentral	5
	5.82	−28	−41	63	L	Postcentral	5
					L	Lingual gyrus	18
					L	Lingual gyrus	18
830	5.54	−13	−79	−12	L	Lingual gyrus	18
	5.45	−7	−87	−17	L	Lingual gyrus	18
	4.15	−7	−87	−22	L	Lingual gyrus	18
	4.13	−7	−82	−14	L	Lingual gyrus	18
	3.82	−3	−88	−11	L	Lingual gyrus	18
486	5.47	−30	−58	57	L	Superior parietal lobule	7
	4.80	−24	−60	56	L	Precuneus	7
	4.62	−22	−67	58	L	Superior parietal lobule	7
	4.55	−33	−51	58	L	Superior parietal lobule	7
	4.24	−25	−64	61	L	Superior parietal lobule	7
	3.94	−17	−67	55	L	Precuneus	7
Negative > Scrambled No cluster FEW < 0.05							
Positive > Negative No cluster FEW < 0.05							
*Group comparison*							
No cluster pFWEc < 0.05							

*Abbr*eviations: *k* cluster size, *t* t scores, *MNI* Montreal Neurological Institute, *BA* Brodman Area

##### Negative emotion processing

Brain activation to negative emotional images was subsequently tested (negative vs. scrambled images). In controls, greater activation to negative images was observed in the right lingual gyrus, bilateral cuneus, and left posterior cingulate. No significant activation was present in participants with 22q11DS, and the direct between-group comparison did not reveal any difference between controls and individuals with 22q11DS, either (see Table 4).

##### Positive versus negative emotion processing

Finally, we contrasted positive vs. negative emotional stimuli to determine regions activated as a function of stimulus valence. Both within- and between-group comparisons did not reveal any significant results.

#### Social content × valence interaction

The social × valence content interaction was tested within and between groups. No significant activations were found.

#### Association with socio-cognitive measures

Finally, we were interested in assessing whether there were any associations between brain activity and socio-cognitive measures in participants with 22q11DS. To do so, we first extracted raw activations (betas) from significant clusters showing up in the analyses (plus a priori ROIs) and tested these betas against face recognition and social functioning scores. No significant associations were observed. Second, we conducted whole-brain multiple-regression analyses with face recognition and social functioning scores. Again, correlations did not reveal any significant effects.

## Discussion

The present study aimed at identifying the neural correlates of socio-emotional perception in individuals with 22q11DS. Results indicated neural hyporesponsiveness within the social perception network in participants with 22q11DS compared to controls. Second, comparable to healthy controls, individuals with 22q11DS showed activation in regions related with emotion processing during the presentation of positive and negative stimuli. No correlation between brain activation and the clinical measures was observed in the 22q11DS population. Finally, results showed no social content × valence interaction differences between patients and controls.

This is the first study investigating the neural correlates of complex social stimuli perception in patients with 22q11DS. In line with our hypothesis, participants with 22q11DS showed atypical patterns of activation during the processing of social information compared to healthy controls. Specifically, we observed reduced brain activation in regions belonging to the default mode network (DMN). Indeed, the DMN encompasses the PFC as the anterior cingulate cortex (ACC) in its anterior part; laterally, it comprises the bilateral IPL and the medial temporal lobes whereas posteriorly it includes the posterior cingulate cortex (PCC) and the precuneus [37, 38]. In the current study, we found hypoactivation of these regions (except for temporal lobes) during the perception of social information in individuals with 22q11DS compared to controls. The DMN is a resting-state network more activated in the absence of a cognitive task and has been well described as being implicated in various socio-cognitive processes such as self-referential processing or theory of mind [39, 40]. Nevertheless, recent literature established that there is an overlap between regions attributed to the DMN and regions activated during socio-cognitive tasks [41, 42]. In disorders characterized by social functioning deficits, such as schizophrenia or autism spectrum disorder (ASD), alterations of the DMN have been previously reported. The majority of studies conducted in schizophrenia points to a decreased functional connectivity of the DMN [43–47], whereas in ASD, altered functional connectivity of DMN regions has been reported during both rest [48–56] and during social tasks [57].

In patients with 22q11DS, alterations of resting state networks, including the DMN, have also been demonstrated using various methods. Whereas studies investigating functional connectivity of multiple resting state networks found hypoconnectivity of the DMN in individuals with 22q11DS [57–59], a multimodal approach revealed both structural and functional connectivity disruption of the DMN [60]. A few studies also examined the behavioural correlates of these alterations. In particular, associations between the strength of the DMN long-range connectivity and social functioning [58] and between a functional decrease of the DMN and prodromal symptom severity have been reported [57].

Our results are in accordance with previous literature in the 22q11DS population and demonstrate a hypoactivation of the DMN during the perception of social information, a basic socio-cognitive process. Taken together, these findings support the involvement of the DMN in socio-cognitive processes and point to a central role of this network in the pathophysiology of 22q11DS. The present study also suggests that alterations of this network could account for social dysfunctions observed in the syndrome. However, it should be noted that post hoc analyses did not reveal any significant correlations between activations within regions of the DMN and the socio-cognitive measures in the group of patients with 22q11DS. Because our sample size is relatively small and we used a non-direct measure of social functioning (parent-reported questionnaire), this finding should be interpreted carefully. Moreover, previous studies showing associations between social functioning and the DMN investigated functional connectivity within the DMN whereas the present study examined BOLD signal. Consequently, further studies investigating the functional connectivity during social perception as well as the association between impaired social perception network and social functioning are required.

Surprisingly, we did not find any significant difference between groups during social perception in the fusiform gyrus, while previous findings found reduced fusiform response during face perception in individuals with 22q11DS [8, 9]. We also failed to see differences in the amygdala and superior temporal sulcus, whereas these two regions appear to be significantly activated during social vs. non-social perception in healthy controls [21]. The lack of result could be due to thresholding differences applied between studies. Indeed, while we applied very strict corrections, previous studies reported results with lower and less-sensitive thresholds [8, 9, 21]. Further studies investigating the activation of these regions during social perception are required.

Taken together, our results clearly indicate alterations in the social perception network in participants with 22q11DS. These alterations could lead to difficulties in social information processing, which could in turn influence various aspects of social cognition and thus contribute to social functioning impairments frequently observed in this syndrome [4–6].

Several explanations could be made to explain differences in the social perception network in 22q11DS. As proposed by Azuma et al. [9], one possible explanation may be a variation in dopamine, which is known to be a neuromodulator of socio-cognitive processes [61]. Individuals with 22q11DS are hemizygous for some genes, including the catechol-O-methyl transferase (COMT) gene that plays a role in dopamine degradation. Consequently, dopamine levels in regions of the social brain could be modified in 22q11DS [62, 63] and lead to altered brain response during social processing. It has already been suggested that dopaminergic genetic variation impacts social perception and behaviour [64]. Indeed, some studies investigating the effects of dopamine variation on ventral striatum response to social reward have demonstrated that heightened dopamine signal is associated with increased response to social reward (impulsive or aggressive) [65, 66] and increased social approach behaviours. Future research investigating the impact of dopamine variation on social perception in 22q11DS is required. Alternatively, these results could also be driven by differences in brain anatomy. Indeed, a previous study conducted in participants with 22q11DS reported a positive relationship between fronto-striatal grey matter volume and social behavioural difficulties [67]. Moreover, structural changes in regions that are part of the social network, such as frontal regions, PCC, and ACC, have already been reported in the 22q11DS literature [68–70]. Thus, larger studies are required to examine the relationship between functional and structural alterations within the social network in the 22q11DS population.

Comparison of the neural correlates of emotion processing within groups revealed that positive stimuli perception was associated with increased activation in the lingual gyrus in both patients and controls. Conversely, increased activation to positive images was found in the PCC and cuneus in controls only, while increased activation in the precuneus, postcentral gyrus, and superior parietal lobule was observed solely in patients. During negative stimuli perception, controls showed greater activation of the right lingual gyrus, bilateral cuneus, and left PCC, while no significant activation was found in patients. Between-group comparisons did not reveal significant difference. These results suggest that during negative and positive emotion processing, patients and healthy controls present a similar pattern of activation. Thus, compared to the social perception network that was clearly atypical in 22q11DS, the neural correlates of emotion perception appear to be preserved in this population. Our results are in contradiction with previous work in the 22q11DS that described hypoactivation of regions related to emotional processing. Indeed, hypoactivation in frontal regions during negative emotional viewing [8, 9] and reduced activation of the insula during mixed emotional faces [7] was found. However, it should be noted that van Amelsvoort et al. reported results from a very small sample (eight patients versus nine controls), so that differences observed between the two studies should be interpreted with caution.

Finally, we examined the influence of emotions on social perception by investigating interactive processing of social content and valence. Our results did not reveal any significant social content × valence interactions either within- or between-group comparisons. Those results are in contraction with previous studies conducted in healthy individuals where a social content × valence interaction emerged [19–21]. Indeed, while Norris et al. [19] and Scharpf et al. [20] reported significant interactions in the thalamus, superior temporal sulcus, and middle orbito-temporal cortex, as well as in the anterior insula and lateral medial prefrontal cortex, Vrtička et al. [21] found such interaction within the amygdala, fusiform, anterior superior frontal gyrus, and middle occipital cortex. One explanation for this absence of social × valence content interaction is a lack of statistical power due the sample size. However, it should be noted that the previous studies reported whole brain or region of interest results with an uncorrected threshold, while we used strict corrections by combining a primary voxel-level statistical threshold at *p* < 0.001 (uncorrected, whole brain) and *k* ≥ 20, with a cluster-extent family-wise correction (FWEc < 0.05). As no differences in interactive processing of social content and valence between groups were found, this suggests that social processing is impaired regardless of the valence in the 22q11DS population.

The results of the present study should be interpreted in light of several limitations. First, we investigated the neural correlates of social perception whereas it is well documented that patients with 22q11DS present visual perception and processing deficits [70, 71]. Indeed, difficulties in object, face, and emotion recognition have been described in the 22q11DS population [71]. Moreover, Magnée et al. [70] reported abnormal transmission between higher and lower visual cortex areas during the presentation of visual stimuli in an event-related potential study. As visual processing deficits could influence social perception, their impact on the observed findings should be further examined. Secondly, although the number of participants included in this study is higher compared to previous work in this field of research [7–9], our sample size remains relatively small. Consequently, results need to be interpreted carefully and studies including a larger sample are required. Thirdly, the neural correlates of social-emotional perception have only been investigated in a cross-sectional way. Thus, the time window during which functional alterations in the social perception network emerge remains unknown. Longitudinal studies examining the developmental trajectory of these alterations in 22q11DS are therefore required. An additional limitation is the potential influence of medication within the group of patients with 22q11DS, which was not investigated in the current study. However, we chose to exclude patients with schizophrenia or another psychotic disorder diagnosis, and only one patient was under antipsychotics at the time of testing. Finally, the neural correlates of additional socio-cognitive processes should be examined in further studies (e.g. theory of mind). Indeed, a better understanding of the neural correlates of social deficits in the 22q11DS population is required.

## Conclusion

The present study provides evidence of specific alterations in the social perception network, irrespective of valence, in the 22q11DS population. Moreover, emotional processing of negative and positive stimuli seems preserved in individuals with 22q11DS. This is the first study investigating lower-order processes of social cognition in the 22q11DS population. The observed alterations are likely to influence higher order socio-cognitive processes. Deficits of social information processing could therefore be a key factor leading to socio-cognitive impairment in the 22q11DS population. Taken together, these findings highlight the need to better understand the emergence of social perception deficits in 22q11DS. Future studies investigating the developmental trajectory of social perception and its association with socio-cognitive processes are required.

## Additional file

Additional file 1: **Table S1.** Characteristic of the IAPS images selected. (DOCX 15 kb)

## Abbreviations

22q11.2DS: 22q11 deletion syndrome
ACC: Anterior cingulate cortex
ASD: Autism spectrum disorder
BFRT: Benton Facial Recognition Test
BOLD: Blood-oxygenation-level-dependent
COMT: Catechol-O-methyl transferase
DARTEL: Diffeomorphic anatomical registration using exponential lie algebra algorithm
DMN: Default mode network
fMRI: functional magnetic resonance imaging
FWEc: Family-wise correction
GLM: General linear model
IAPS: International Affective Picture System
IPL: Inferior parietal lobule
K-SADS-PL: Kiddie-Schedule for Affective Disorders and Schizophrenia Present and Lifetime version
MNI: Montreal Neurologic Institute
PCC: Posterior cingulate cortex
QF-PCR: quantitative fluorescent polymerase chain reaction
ROI: Region of interest
SCID-I: Structured Clinical Interview for DSM-IV axis disorders
SIPS: Structured Interview for Psychosis-risk Syndromes
SPM12: Statistical Parametric Mapping 12
SRS: Social Responsiveness Scale
VCFS: Velocardiofacial syndrome
WAIS III: Wechsler Adult Intelligence Scale, third version
WAIS IV: Wechsler Adult Intelligence Scale, fourth version
WISC III: Wechsler Intelligence Scale for Children, third version
WISC IV: Wechsler Intelligence Scale for Children, fourth version
