# Supplementary material for: Neural correlates of socio-emotional perception in 22q11.2 deletion syndrome
Source: J Neurodev Disord. 2018 Apr 10;10:13. doi: 10.1186/s11689-018-9232-2 (PMC5891973; doi:10.1186/s11689-018-9232-2)
Supplement: Supplementary file 1 — Table S1. Characteristic of the IAPS images selected. (DOCX 15 kb) [file 11689_2018_9232_MOESM1_ESM.docx]

**Additional file 1: Table 1.** Characteristic of the IAPS images selected

| **Condition** | **Social positive** | **Non-social positive** | **p-value*** | **Social negative** | **Non-social negative** | **p-value*** |
| --- | --- | --- | --- | --- | --- | --- |
| **Valence (Mean ± SD)** | 75.3 (4.6) | 76.9 (6.0) | **0.283** | 20.9 (4.9) | 21.5 (4.1) | **0.713** |
|  |  |  |  |  |  |  |
| **Arousal (Mean ± SD)** | 43.3 (6.3) | 43.2 (5.3) | **0.921** | 70.3 (3.9) | 68.6 (4.5) | **0.224** |

**** T-test comparison***

**Group comparison without IQ as covariate**

Group comparison of the main contrast of interest, without including IQ as covariate point out similar findings. Indeed, the social vs. non-social contrast revealed greater activation in controls compared to 22q11DS patient in the following DMN regions: right precuneus, bilateral inferior parietal lobule as well as in the right anterior cingulate cortex. Controls exhibit also higher activation in right superior parietal lobule, left precentral gyrus, left postcentral gyrus and left inferior occipital gyrus in comparison to 22q11DS individuals. For the emotional contrast, (positive vs. negative image) comparison between groups did not revealed significant result.
